# Supplementary material for: Happy software developers solve problems better: psychological measurements in empirical software engineering
Source: PeerJ. 2014 Mar 11;2:e289. doi: 10.7717/peerj.289 (PMC3961150; doi:10.7717/peerj.289)
Supplement: Article S1 [file peerj-02-289-s001.pdf]

Happy software developers solve problems better

Reference Sheet for the experiment participants.

Daniel Graziotin, Xiaofeng Wang, Pekka Abrahamsson
Faculty of Computer Science, Free University of Bozen-Bolzano, Italy
{first.last}@unibz.it

Hi and thank you for participating to this experiment. This sheet contains the instructions for completing it. First, please do not logout/shutdown/reboot the PC. We will lose your data otherwise.

Your Reference Code is: **<Reference Number>**

Please, provide it when requested. The experiment is completely anonymous. We only need the Reference Code to connect your surveys with the data that you will provide us during the experiment.

If you have a question, feel free to call one of the supervisors whenever you want.

The following are the experiment phases.

1. Survey

Please open the browser and go to <URL> to reach the survey. Answer to all the provided questions. Remember that the period is the past 4 weeks, including right now. Provide the Reference Code **<Reference Number>**.

Remember to submit the Survey once you have finished it. It should take you less than 5 minutes to complete it, but take your time.

2. Photographs game

Go to the supervisors and provide your Reference Code **<Reference Number>**. You will receive two photographs, one at a time. Imagine that you are participating in the Best Caption of the Year contest. This contest is organized by a famous magazine and the winning captions will be published along with the photographs. Your job is to try to win this contest by writing the best captions possible for each of these two photographs. The captions can be absolutely anything you would like. You can write as many captions as you would like. Please, remember to write your Reference Code **<Reference Number>** in the photographs, too. This task should take you less than 20 minutes, but take your time.

3. Survey

Please open the browser and go to <URL> to reach the survey. Answer to all the provided questions. Remember that the period is the past 4 weeks, including right now. Provide the Reference Code **<Reference Number>**.

Remember to submit the Survey once you have finished it. It should take you less than 5 minutes to complete it, but take your time.

4. Tower of London game

Please open the PEBL software. As Participant Code (located in the top-center section of the user interface), enter your Reference Code **<Reference Number>**. Do not press the "+" button.

On the left side panel, follow this path: *battery/ → tol/ → TOL.pbl*. Select TOL.pbl with the mouse. Click the button labeled "Add to Chain". The TOL.pbl will appear in the Experiment Chain list. Click the button labeled "Launch Chain". A new window will appear. When requested, press key 3 on the keyboard to select Shallice Test ([1, 2, 3] pile heights, 3 disks, Shallice's 12 problems).

It should take you about 10 minutes to finish the game, but take your time.

When you finish the game, please call one of the supervisors of the experiment. Do not close the program. We remember you again; please do not logout/shutdown/reboot the PC.

Thank you for your collaboration.
