# Supplementary figures and images for: Happy software developers solve problems better: psychological measurements in empirical software engineering
Source: PeerJ. 2014 Mar 11;2:e289. doi: 10.7717/peerj.289 (PMC3961150; doi:10.7717/peerj.289)

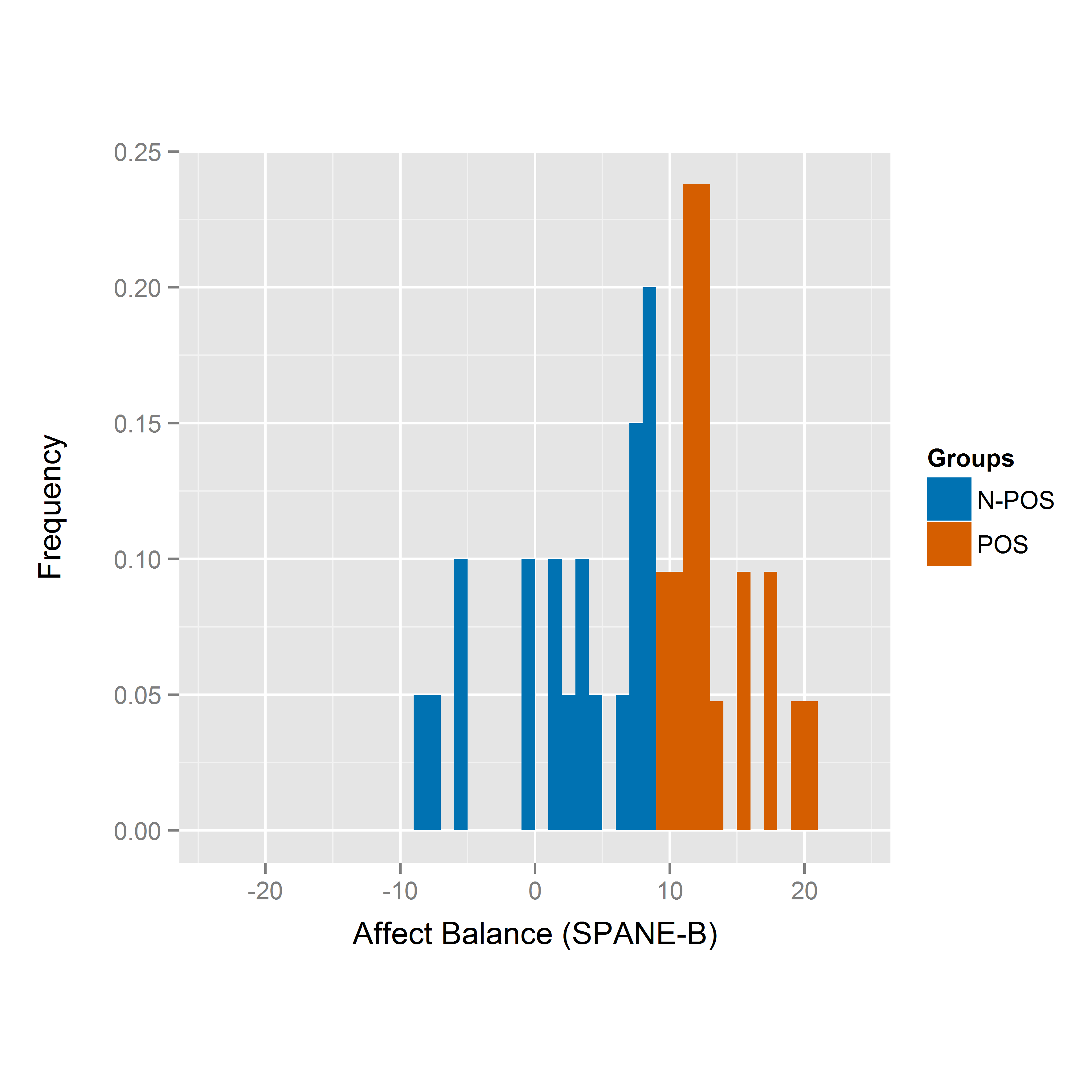

Supplement: Figure S1 [file peerj-02-289-s002.png]

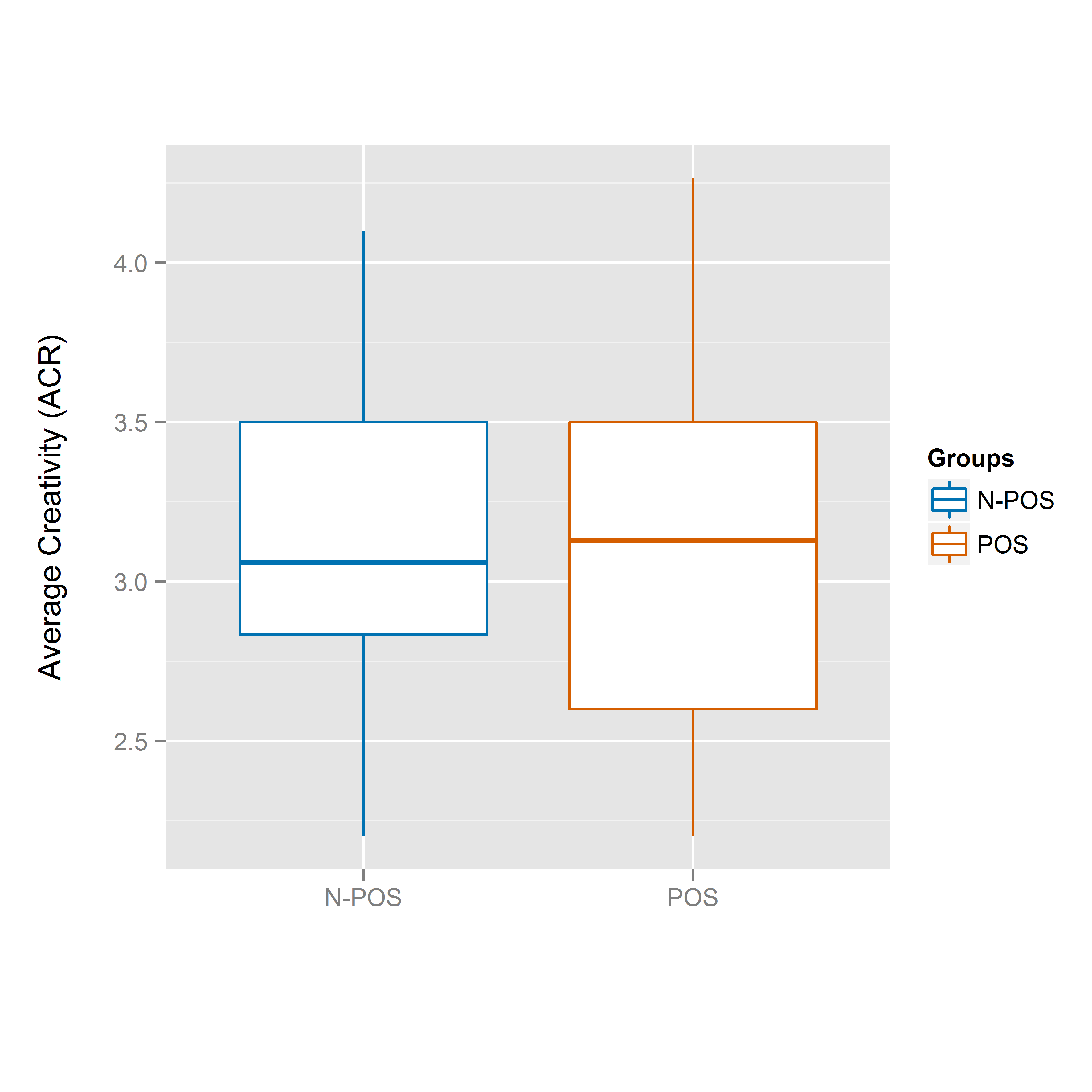

Supplement: Figure S2 [file peerj-02-289-s003.png]

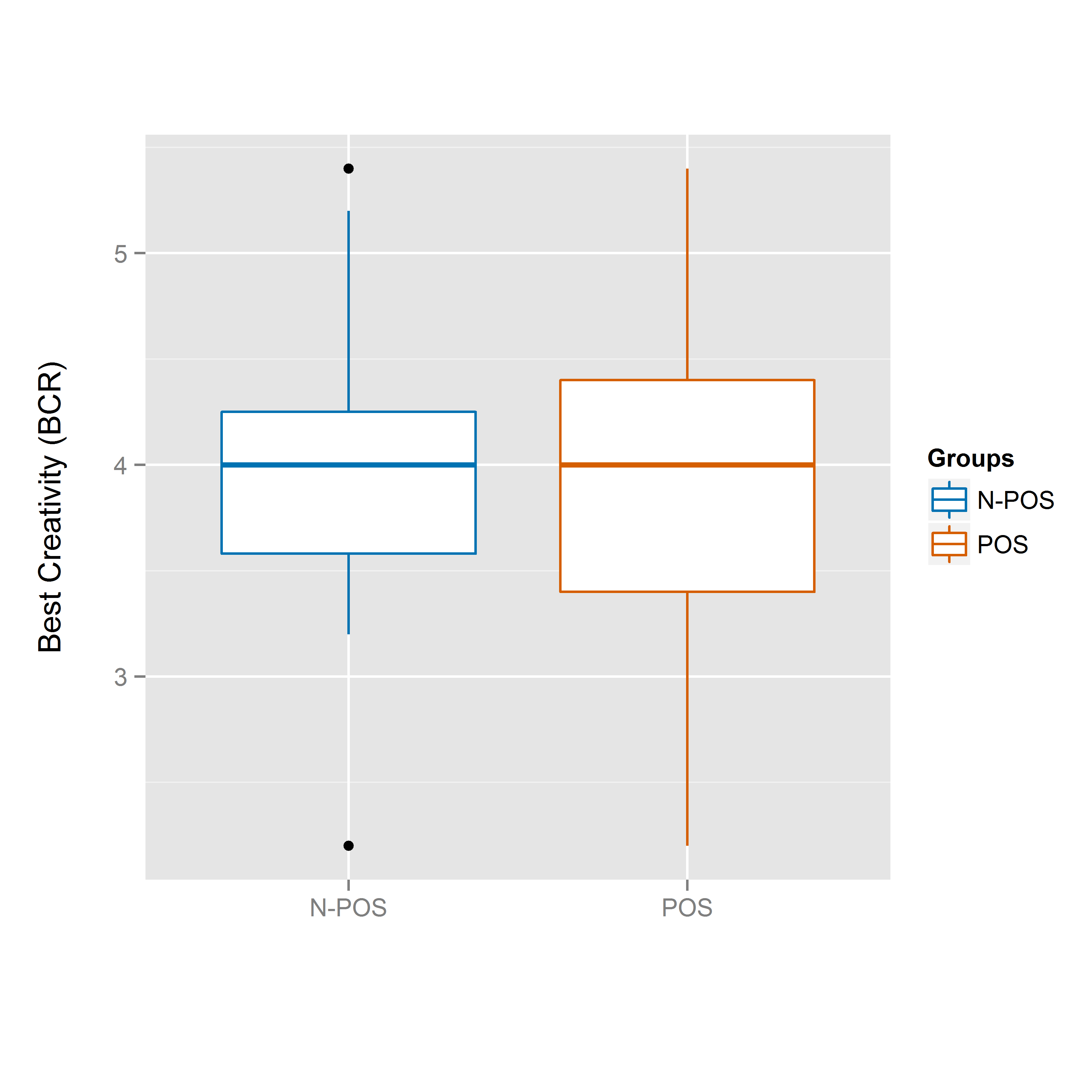

Supplement: Figure S3 [file peerj-02-289-s004.png]

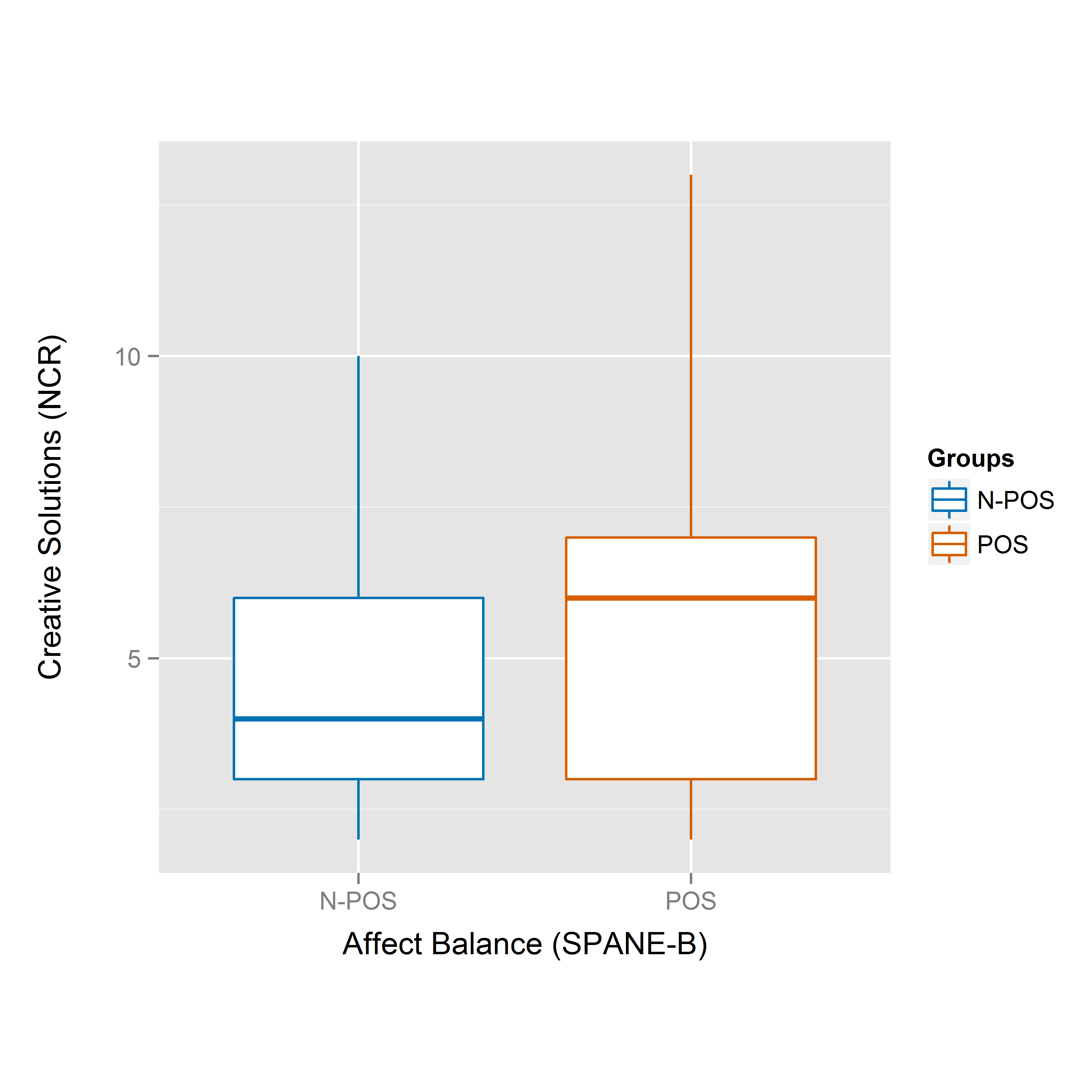

Supplement: Figure S4 [file peerj-02-289-s005.png]

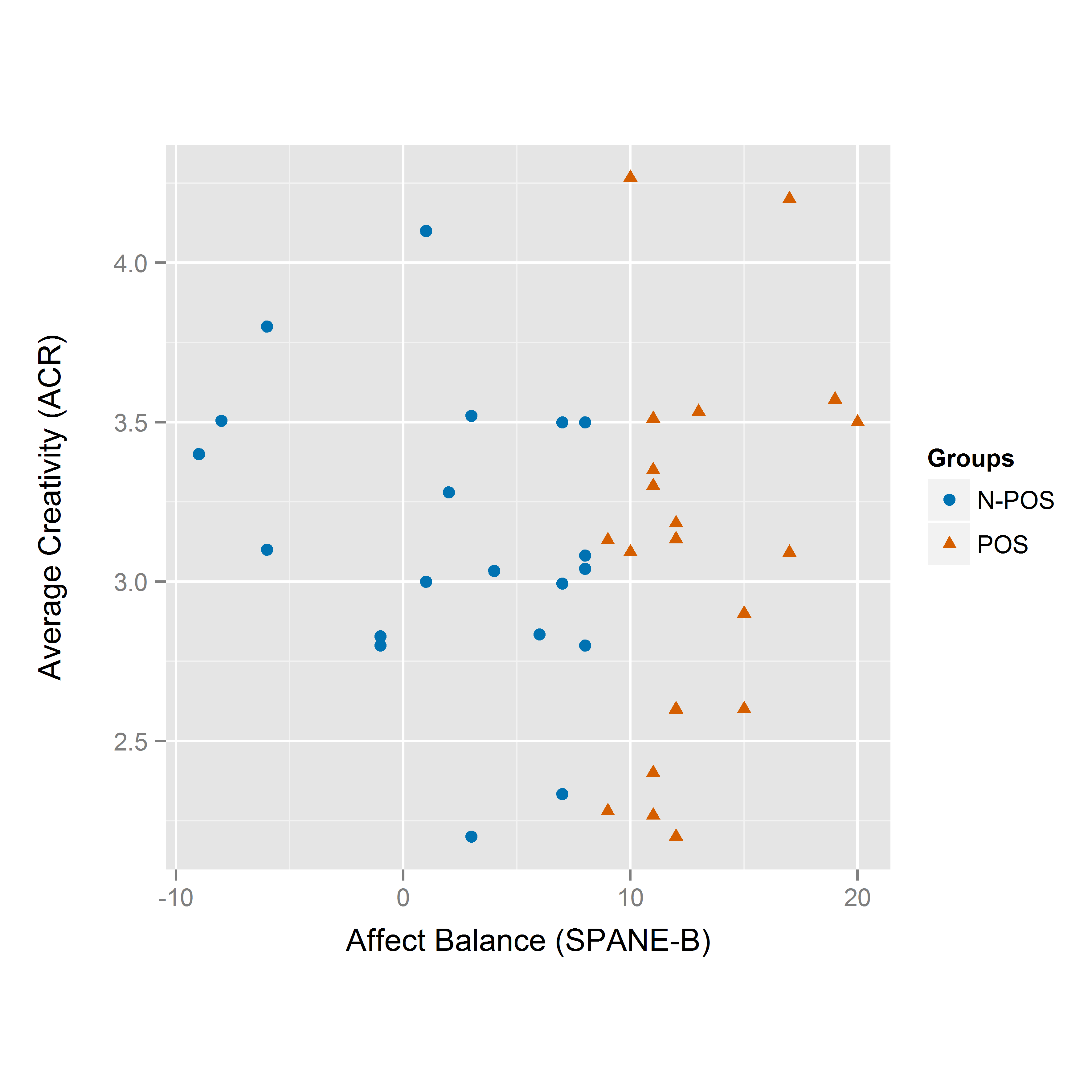

Supplement: Figure S5 [file peerj-02-289-s006.png]

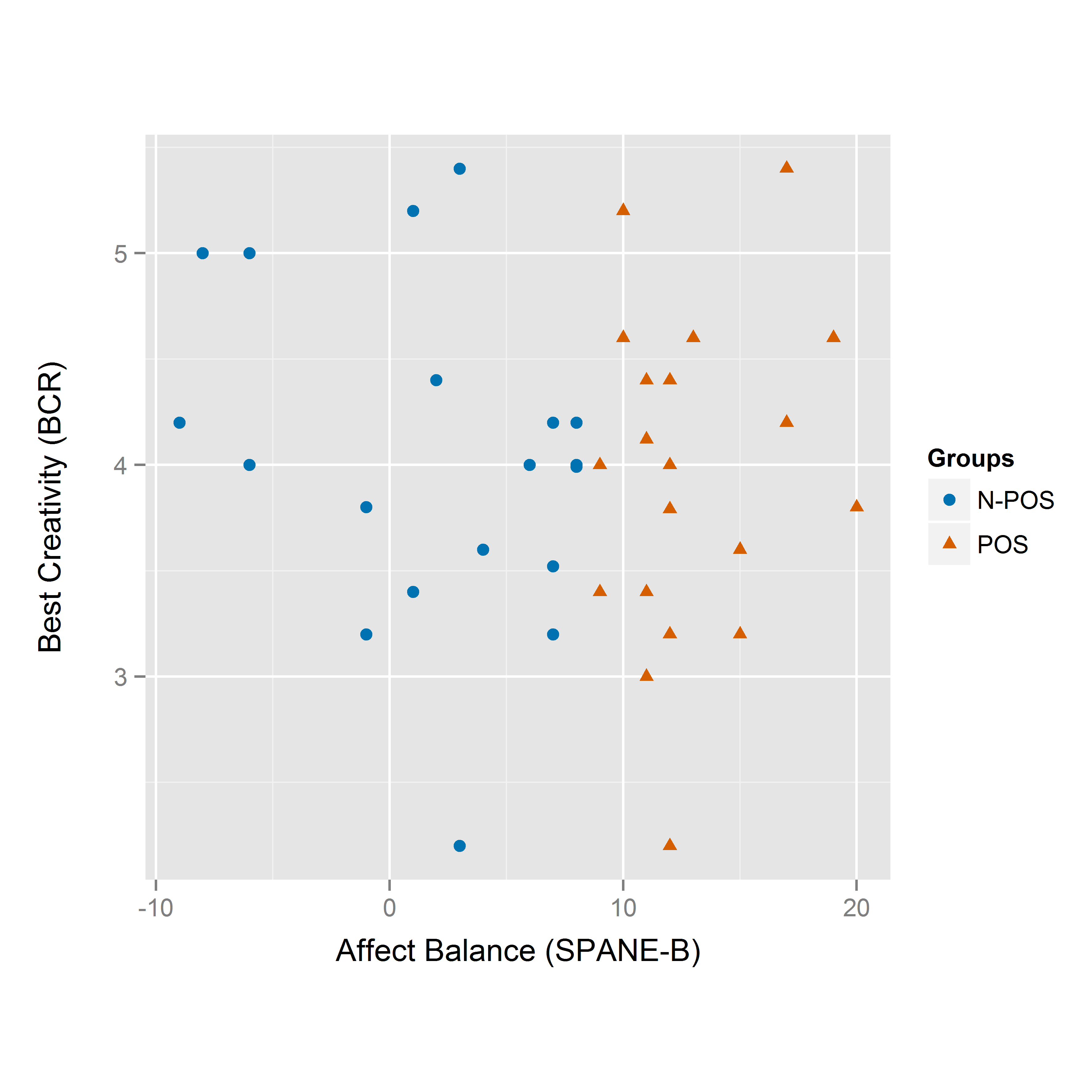

Supplement: Figure S6 [file peerj-02-289-s007.png]

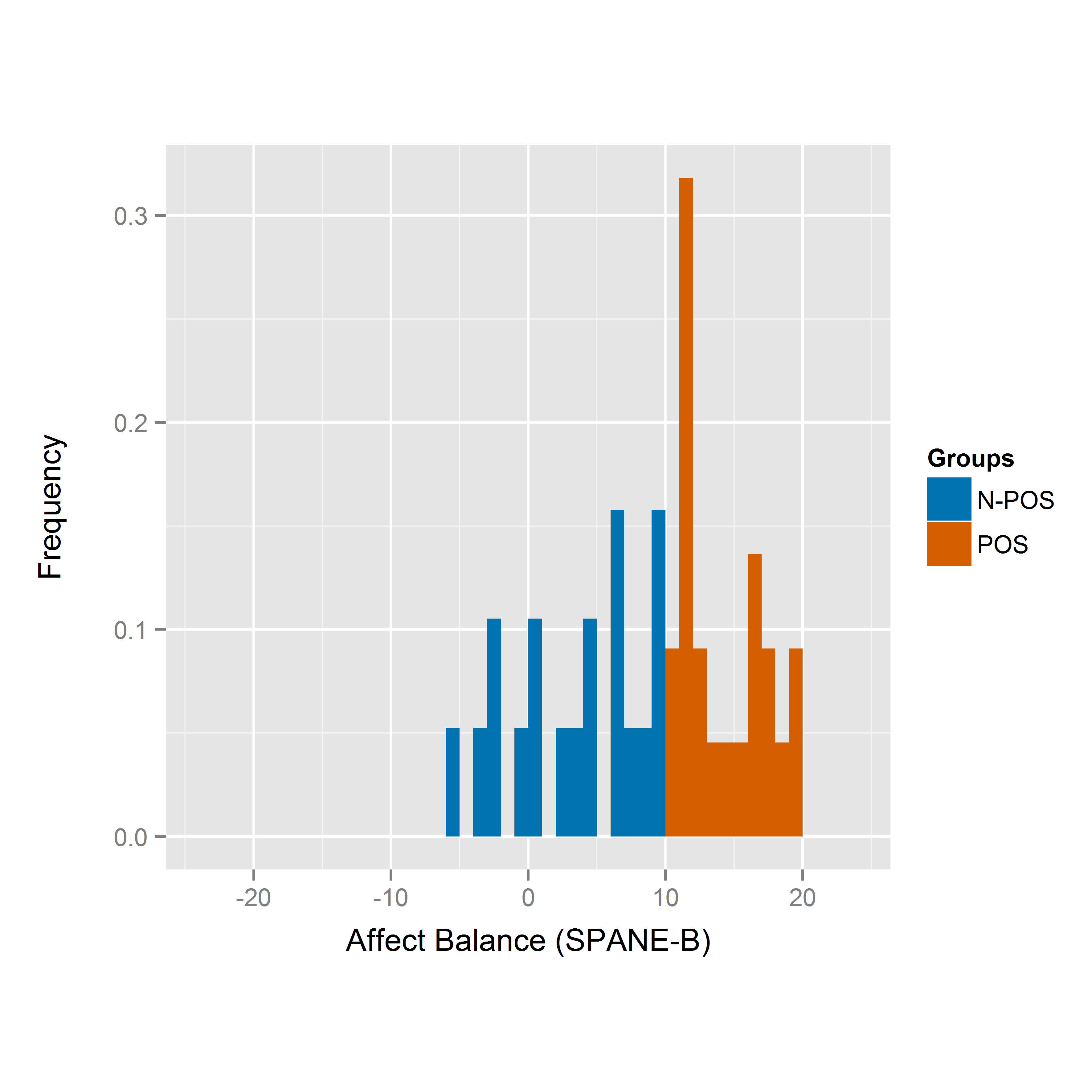

Supplement: Figure S7 [file peerj-02-289-s008.png]
